# Policy recommendations to reduce high cesarean section rate in Türkiye

**DOI:** 10.11604/pamj.2024.48.83.11726

**Published:** 2024-07-02

**Authors:** Mert Küçük

**Affiliations:** 1 Department of Obstetrics and Gynecology, Mugla Sitki Kocman University, Faculty of Medicine, 48000 Menteşe, Muğla, Türkiye,; 2 Department of Medical Education and Bioinformatics, Mugla Sitki Kocman University, Faculty of Medicine, 48000 Menteşe, Muğla, Türkiye

**Keywords:** Policy recommendation, Türkiye, health transformation program, cesarean section

## Abstract

According to the latest data, the majority of babies are delivered via cesarean section (C-section) in Türkiye. This is a medically unacceptable situation and reflects the highest rate in the world. Despite Türkiye enacting a law prohibiting elective C-sections, the rate has not decreased; rather increased. Therefore, this article and accompanying annex have been prepared to propose policy recommendations aimed at reducing the C-section rate. This article summarizes what has been done so far and outlines areas for improvement to reduce the C-section rate, offering a different perspective on policy. The C-section rate has reached such a critical level in Türkiye that a concerted effort involving the Turkish Ministry of Health, healthcare and obstetrics societies, physicians, midwives, healthcare professionals, public and the media is necessary. Health authorities in Türkiye should consider all policy recommendations in addressing this issue.

## ESSAY

The health transformation program, which was initiated in 2003, aimed to improve healthcare outcomes in Türkiye. However, it inadvertently led to a drastic increase in cesarean section (C-section) rate, surpassing Organization for Economic Cooperation and Development (OECD) averages and global benchmarks. In 1988, Turkey had a C-section rate of 5.7% that rose to 7% by 1993, rising further to 14% by 1998. In 2006, the rate reached 31.7%, and by 2010, it escalated to 47.2%. Subsequently, in 2014, it rose to 51%, and by 2021, it had reached 58.4% [1]. This introduction highlights the significance of the issue and the challenges posed by excessively high C-section rate. On July 4, 2012, the Turkish parliament enacted a legislation aimed at restricting C-sections to medically necessary cases only. Türkiye was reportedly the first country to introduce penalties, including fines, for doctors performing elective C-sections. According to Article 153 of the Public Health Act, C-sections are permitted only if there is a medical necessity for the pregnant woman or the fetus [2]. The C-section rate in Türkiye has surged significantly since the implementation of the health transformation program in 2003, reaching the world´s highest. Despite efforts by the Turkish Ministry of Health (TMOH), including legislative measures to ban elective C-sections, the rate continues to rise [1].

This article outlines recommendations to reverse this trend, emphasizing collaboration among health authorities, obstetrics societies, healthcare professionals, public and the media.

**Current situation:** Türkiye now faces the highest C-section rate globally, with significant regional disparities.

**Factors behind high C-section rates:** various factors which includes medical practices, cultural preferences, and institutional factors contribute to high C-section rates.

**Health implications:** the high prevalence of C-sections in Türkiye poses significant medical risks, including increased maternal and neonatal complications and morbidity.

**Policy measures:** TMOH´s efforts are summarized in the paper and in the accompanying annex which includes legislative actions and health campaigns. Despite legislation aimed for decreasing elective C-sections, rate continues to rise, which highlights the need for comprehensive policy interventions. This article and the accompanying annex summarize what has been done so far and outline areas for improvement to reduce high C-section rate, offering a different perspective on policy.

C-section serves as a life-saving procedure in obstetric practice when used in indicated conditions. With the development of anesthetic services, the use of antibiotics, and advancements in surgical techniques, significant reductions in morbidity and mortality related to C-section have been achieved [3]. Especially in the last decade, C-sections have become more common procedures in both developed and developing countries. Today, one of the most performed surgical operations worldwide is the C-section [2]. In recent years, there has been a rapid increase in the C-section rates. While high C-section rates are reported from various countries, the data from Türkiye is particularly alarming. According to the latest data, the majority of babies are born via C-section in Türkiye [1]. This situation is medically unacceptable and reflects the highest rate in the world [4]. It should be noted that the overuse of C-sections is associated with serious life-threatening and unwanted conditions that can affect future pregnancies. Unless the C-section rate is reduced, the negative impacts will eventually emerge, leading to increased complications and, consequently, maternal and neonatal mortality. For example, placenta previa will increase in recurrent pregnancies, potentially leading to life-threatening placenta accreta [5].

Despite Türkiye enacting a law forbidding elective C-sections, the rate has not decreased, rather increased. Therefore, this article and accompanying annex have been prepared to suggest policy recommendations to reduce the C-section rate. This article summarizes what has been done so far and identifies areas for improvement to reduce the C-section rate, offering a different perspective on policy. The C-section rate has reached such a critical level in Türkiye that firm cooperation of the TMOH, healthcare and obstetric societies, physicians, midwives, healthcare professionals, and media is necessary. Health authorities in Türkiye should take all kinds of policy recommendations into account [2].

**Doula support:** in Annex 1 (item 1), the concept of doula support is highlighted. Numerous studies have demonstrated the effectiveness of doula support [6]. TMOH should prepare a detailed roadmap for doula support and declare this roadmap to health professionals and the public. Widespread implementation of doula support as a state policy could impact the high C-section rate prevalent in Türkiye.

Determining the optimal C-section rate for Türkiye and monitoring of C-section rates: there are complexities in determining an appropriate C-section rate tailored to Türkiye's healthcare landscape while acknowledging global recommendations. Monitoring C-section rates in various settings is crucial (Annex 1 - item 2). Multiple factors contribute to the rising C-section rate, making it challenging to pinpoint a single cause. Similarly, advocating for an optimal C-section rate is complex. Although health authorities in Türkiye strongly emphasize a 15% C-section rate, recommended by the World Health Organization, this rate may not be realistic for Türkiye's unique healthcare landscape [2,7]. Turkish Ministry of Health (TMOH) currently monitors institutions for C-section rates, promotes the use of Robson classification nationwide, and publishes annual C-section rates; however, improvements are needed in ensuring meticulous completion of Robson classification forms at all health institutions and addressing the educational requirements of obstetricians and other healthcare staff regarding this classification system. Further discussions on optimal C-section rates and monitoring of C-section rates are included in Annex 1 (item 2-3).

**Supporting high-quality research:** interdisciplinary research to identify and address factors contributing to high C-section rates, involving stakeholders across healthcare and policymaking domains are crucial. Reducing the C-section rate hinges on understanding the reasons behind unnecessary C-sections. It is imperative to examine these reasons from the perspectives of all stakeholders, including obstetricians, midwives, patients, healthcare managers, and policymakers. Turkish Ministry of Health (TMOH) should develop a comprehensive action plan to address the alarmingly high C-section rate, incorporating input. Timely data collection, robust management infrastructure, and interdisciplinary collaboration involving midwives, obstetricians, and other healthcare professionals should be prioritized by this plan. Continuous monitoring and refinement of the plan are essential, with a primary focus on understanding the underlying reasons for the high C-section rate. Supporting high-quality research (Annex 1 - Item 4) is crucial for planning evidence-based interventions aimed at reducing unnecessary C-sections. Further discussions on this topic are included in Annex 1.

Increasing awareness, birth preparedness training and overcoming tokophobia: enhancing public awareness and evidence-based birth preparedness training is critical. Targeted media campaigns can promote informed decision-making among expectant mothers. A crucial requirement is providing accurate information to the public regarding pregnancy, labor, and birth [8]. Women of childbearing age and their families should have access to evidence-based medical data related to childbirth. Normal vaginal delivery, supported by evidence-based medicine, offers significant advantages. Disseminating this knowledge widely among women of childbearing age can positively influence their behaviors and attitudes. Organizing awareness campaigns in the media on this issue is of critical importance (Annex 1 - item 5). While the TMOH has initiated such campaigns, they have generally been ineffective. Therefore, it is essential to clearly define the objectives of these campaigns and tailor them to specific target groups. Effective campaigns should be developed in collaboration with women's organizations and civil society groups, with periodic evaluations of their effectiveness. Evaluations should include assessments of the knowledge, attitudes, and behaviors of target groups such as women of childbearing age, pregnant women, midwives, and obstetricians, using consistent and valid instruments. Pregnancy schools (birth preparedness training) and interventions to address pregnant women's fear of labor pain have the potential to reduce C-section rate and are discussed in Annex 1 item 6, and Annex 1 - item 7). The content and effectiveness of education in pregnancy schools should be assessed regularly [9].

**Meeting training needs of healthcare staff:** strengthening ongoing education and training programs for healthcare professionals to enhance skills in C-section-reducing interventions and reduce reliance on C-sections is important. The training needs of healthcare staff should be addressed in this regard. Continuous postgraduate training for midwives and obstetricians should be conducted regularly, focusing on procedures such as instrumental delivery (ID) and external cephalic version (ECV) which have the potential lower the C-section rate [10]. However, these maneuvers are gradually being phased out and are often excluded from the training of gynecology and obstetrics assistants. It is imperative to thoroughly evaluate and improve the training programs for gynecology and obstetrics assistants, obstetricians, and midwives. This includes enhancing the content, quality, and duration of training programs to ensure that healthcare professionals are equipped with the necessary skills and knowledge to effectively reduce the C-section rate (Annex 1 - items 8-10).

**Medical laws and regulations:** there is a need for reform in medical litigation laws, particularly to alleviate the increased fear among obstetricians regarding legal repercussions that arose after the enactment of the new Turkish Penal Code in 2005, and to encourage adherence to evidence-based birthing practices. One of the significant reasons attributed to the high C-section rate is the fear of medical litigation among obstetricians. In recent years, defensive medicine practices among obstetric staff have been linked to the high C-section rate. Obstetricians often opt for C-sections, considered safer, due to the fear of potential high compensations in cases of normal birth [11,12]. Consequently, interventions like ID and ECV are often avoided despite their potential to reduce the C-section rate [13]. To address these issues, regulations compatible with the realities of Türkiye must be enacted to encourage healthcare staff to reduce the high C-section rate. This could include establishing medically specialized courts and enacting specific medical malpractice laws. Medico-legal considerations related to the high C-section rate are thoroughly assessed in Annex 1 (items 11-13).

**Empowering midwives:** empowering midwives in delivery settings and pregnancy follow-ups has the potential to reduce C-section rate. In countries with low C-section rates, midwives play a more significant role in delivery, labor, and pregnancy follow-ups [14]. Turkish Ministry of Health (TMOH) should support initiatives aimed at giving midwives greater authority and responsibility within this context (Annex 1 - item 14).

Improving healthcare personnel and delivery room infrastructure: improving healthcare personnel and delivery room infrastructure is an important step to reduce C-section rate. Although progress has been made in healthcare personnel and delivery settings since the inception of the health transformation program, there are areas that require improvement (Annex 1 - items 15, 16). Enhancing the number and quality of healthcare personnel and improving delivery room infrastructure has the potential to reduce the high C-section rate.

**Implementing reinforcement strategies:** while the TMOH has applied negative reinforcements, their effectiveness seems limited. Encouraging positive reinforcements and conducting further assessment of the high C-section rate in private hospital settings are imperative (Annex 1 - items 17, 18).

**Other strategies:** other practices with the potential to reduce C-section rate in Türkiye are summarized in the accompanying annex.

## Conclusion

Despite stringent measures, the C-section rate in Türkiye continues to rise, highlighting the complexity of the issue beyond legislative interventions. There is a critical need for a comprehensive, multifaceted approach to effectively manage and reduce C-section rate while ensuring maternal and fetal health. Reducing Türkiye's high C-section rate requires a concerted effort involving all stakeholders. This policy brief proposes a reevaluation of current strategies and recommends policy strategies to address the issue effectively. Implementing these policy recommendations can mitigate medical risks associated with unnecessary surgeries and improve maternal and neonatal outcomes.

**Implications and recommendations:** health authorities should prioritize the adoption of doula support, facilitate discussions on optimal C-section rate, support research initiatives, enhance effective birth preparedness training, improve healthcare staff training, and reform medical laws alongside with other proposed strategies in the current paper. Collaborative efforts are essential in achieving sustainable reductions in C-section rate and ensuring safer childbirth practices in Türkiye.

## Annex

**Annex 1****:** policy recommendations to reduce cesarean sections (PDF-124KB)

## References

[1] OECD data Caesarean sections.

[2] Küçük M (2024). Striking rise of cesarean section rates in Türkiye: there is a need for a whole new perspective. Pan Afr Med J.

[3] Arjun G (2008). Caesarean section: evaluation, guidelines and recommendations. Indian J Med Ethics.

[4] Ulgu MM, Birinci S, Altun Ensari T, Gözükara MG (2023). Cesarean section rates in Türkiye 2018-2023: Overview of national data by using Robson ten group classification system. Turk J Obstet Gynecol.

[5] Silver RM, Landon MB, Rouse DJ, Leveno KJ, Spong CY, Thom EA (2006). Maternal morbidity associated with multiple repeat cesarean deliveries. Obstet Gynecol.

[6] Hodnett ED, Gates S, Hofmeyr GJ, Sakala C (2013). Continuous support for women during childbirth. Cochrane Database Syst Rev.

[7] World Health Organization (1985). Appropriate technology for birth. Lancet.

[8] British Columbia Perinatal Health Program (2008). Caesarean Birth Task Force. Caesarean Birth Task Force Report 2008.

[9] Taşpınar A, Özpınar S, Çoban A, Küçük M (2014). The effects of prenatal care on cesarean section rates in a maternity and children's hospital. Cumhuriyet Med J.

[10] ACOG Committee on Obstetric Practice (2006). ACOG Committee Opinion No. 340. Mode of term singleton breech delivery. Obstet Gynecol.

[11] Küçük M (2017). Obstetrician perceptions of the causes of high cesarean delivery rates in Turkey. Int J Gynaecol Obstet.

[12] Küçük M (2018). Defensive medicine among obstetricians and gynaecologists in Turkey. J Obstet Gynaecol.

[13] Clock C, Kurtzman J, White J, Chung JH (2009). Cesarean risk after successful external cephalic version: a matched, retrospective analysis. J Perinatol.

[14] Josefsson A, Gunnervik C, Sydsjö A, Sydsjö G (2011). A comparison between Swedish midwives' and obstetricians' & gynecologists' opinions on cesarean section. Matern Child Health J.

